# Difference in Antibody Responses to* Mycobacterium tuberculosis* Antigens in Japanese Tuberculosis Patients Infected with the Beijing/Non-Beijing Genotype

**DOI:** 10.1155/2017/4797856

**Published:** 2017-01-15

**Authors:** Jingge Zhao, Beata Shiratori, Masao Okumura, Hideki Yanai, Makoto Matsumoto, Chie Nakajima, Kazue Mizuno, Kenji Ono, Tetsuya Oda, Haorile Chagan-Yasutan, Yugo Ashino, Takashi Matsuba, Takashi Yoshiyama, Yasuhiko Suzuki, Toshio Hattori

**Affiliations:** ^1^Division of Emerging Infectious Diseases, Department of Internal Medicine, Graduate School of Medicine, Tohoku University, Sendai, Miyagi 980-8574, Japan; ^2^Laboratory of Disaster-Related Infectious Disease, International Research Institute of Disaster Science, Tohoku University, Sendai, Miyagi 980-8574, Japan; ^3^Fukujuji Hospital, Japan Anti-Tuberculosis Association, 3-1-2 4 Matsuyama, Kiyose, Tokyo 204-8533, Japan; ^4^Microbiological Research Institute, Otsuka Pharmaceutical Co., Ltd., Kawauchi-cho, Tokushima 771-0192, Japan; ^5^Division of Global Epidemiology, Research Center for Zoonosis Control, Hokkaido University, Sapporo, Hokkaido 001-0020, Japan; ^6^Division of Bacteriology, Department of Microbiology and Immunology, Faculty of Medicine, Tottori University, Yonago, Tottori 683-8503, Japan; ^7^Graduate School of Health Science Studies, Kibi International University, 8 Igamachi, Takahashi 716-8508, Japan

## Abstract

The Beijing genotype* Mycobacterium tuberculosis* (MTB), notorious for its virulence and predisposition to relapse, could be identified by spoligotyping based on genetic heterogeneity. The plasma samples from 20 cases of Beijing and 16 cases of non-Beijing MTB infected individuals and 24 healthy controls (HCs) were collected, and antibodies against 11 antigens (Rv0679c142Asn, Rv0679c142Lys, Ag85B, Ag85A, ARC, TDM-M, TDM-K, HBHA, MDP-1, LAM, and TBGL) were measured by ELISA. Compared to the HCs, the MTB infected subjects showed higher titers of anti-Ag85B IgG (positivity 58.2%) and anti-ACR IgG (positivity 48.2%). Of note, anti-ACR IgG showed higher titer in Beijing MTB infected tuberculosis (TB) patients than in HC (Kruskal–Wallis test, *p* < 0.05), while the levels of anti-Ag85B, anti-TBGL, anti-TDM-K, and anti-TDM-M IgG were higher in non-Beijing TB patients than in HC. Moreover, anti-Ag85B IgG showed higher response in non-Beijing TB patients than in Beijing TB patients (*p* < 0.05; sensitivity, 76.9% versus 44.4%). The sensitivity and specificity analysis showed that 78.8% Beijing infected individuals were negative in anti-TBGL-IgG or/and anti-Ag85B-IgG, while 75.0% of those were positive in anti-TBGL-IgA or/and anti-ACR-IgG tests. These results indicate the possibility of developing antibody-based test to identify Beijing MTB.

## 1. Introduction

In 2013, tuberculosis (TB) infected 9 million individuals and caused 1.5 million deaths, making it one of the most critical infectious diseases worldwide [1]. Beijing genotype MTB has been most prevalent in East Asia [2], because of its virulence and resistance to chemical drugs and BCG vaccination [3]. Unfavorable treatment outcomes, including treatment failure and relapse, have also been found to be associated with the Beijing genotype MTB [4, 5]. Genotyping methods, such as spoligotyping and variable number tandem repeats typing (VNTR), have been commonly used to identify MTB genotypes, on the basis of polymerase chain reaction (PCR) [6, 7]. However, the sensitivity of these tests pertains to acid-fast bacillus (AFB) smear results as low-AFB-positive samples (≤1+) showed limited sensitivity of <50% [8], and DNA-negative samples did not yield spoligopatterns [9]. This may be complemented by sputum culture, which may take 1–8 weeks for results at best [7]. Therefore, point-of-care testing (POCT) methods such as ELISA may help in quick differentiation of cases of Beijing and non-Beijing genotype MTB.

*Rv0679c* sequence is well conserved in MTB,* M. bovis*, and* M. bovis* BCG. The Rv0679c protein is a possible membrane lipoprotein located at the bacterial outer surface that may be involved in the entry of MTB into host cells [10, 11]. Beijing genotype MTB carries a specific mutation on the* Rv0679c* gene, causing amino acid replacement at codon 142 from asparagine to lysine (Rv0679c142Lys) in contrast to the non-Beijing genotype MTB (Rv0679c142Asn) without this mutation [12]. Both TB Antigen 85A (Ag85A) and Antigen 85B (Ag85B) are involved in mycobacterial cell wall assembly, possessing a mycolyl-transferase activity that plays a crucial role in the biogenesis of trehalose dimycolate (TDM) [13]. Ag85A may mainly facilitate MTB to survive in minimal nutritional medium [13], whereas Ag85B is reported as a potent immunoprotective antigen and a leading drug target [14, 15]. Of note, Ag85B expression was reported to be lower in the Beijing/W lineage strains compared to the non-Beijing/W lineage strains [16, 17]. The 16-kDa *α*-crystalline (ACR) protein of MTB, also known as HspX or Hsp 16.3, is required for MTB growth in macrophages [18] and is highly expressed in the presence of NO and low O_2_ concentration and in the stationary phase [19, 20], playing a role in the maintenance of long-term viability during latent, asymptomatic infections [18], which was found to be highly expressed in the Beijing group [16]. The heparin-binding hemagglutinin adhesin (HBHA) is indispensable for extrapulmonary dissemination [21], while the Beijing genotype MTB is more likely to develop extrapulmonary TB [22]. The surplus expression of mycobacterial DNA-binding protein 1 (MDP-1) in the slow-growing phase is a potential marker for latent TB infection (LTBI) for both non-Beijing and Beijing MTB [23]. TDM derivatives, including trehalose methoxy-dimycolate (TDM-M) and trehalose keto-dimycolate (TDM-K), from MTB H37Rv constitute the major antigens of TBGL [24]. Anti-TBGL IgG and anti-lipoarabinomannan (LAM) antibodies were tested in the clinic as biomarkers for active TB infections (ATB) [25]. In our previous study, high plasma levels of anti-ACR, anti-LAM, anti-trehalose dimycolate (anti-TDM), and anti-TBGL IgG were found in patients with ATB, compared to those in the LTBI and control subjects [26]. Here we investigated the possibility of serological identification of Beijing/non-Beijing genotype MTB by using an in-house ELISA method.

## 2. Materials and Methods

### 2.1. Study Subjects

This study was approved by the Ethics Committee of Tohoku University and Fukujuji Hospital, number* 2014-1-122*. Signed informed consent was obtained from all study participants. All ATB subjects were diagnosed following the WHO guidelines on TB at Fukujuji Hospital, Japan. For TB patient samples, only culture-positive samples were included. Blood samples were collected and stored before initiating anti-TB therapy, to avoid the effects of decreasing antibody titer during therapy [27]. Plasma samples were stored at −30°C immediately after extraction without experiencing the repeated freeze-thaw cycle prior to the test. Twenty-six HCs were evaluated for LTBI by the T-SPOT test (Oxford Immunotec, Inc., MA), in which 24 of them (92.3%) were tested as negative; they were recruited in this study. In total, the plasma samples of 36 TB patients and 24 HCs were tested under a double-blind study in which the results of spoligotyping test accomplished by Dr. Chie Nakajima and the results of ELISA test accomplished by Dr. Jingge Zhao were handed to the corresponding author Dr. Toshio Hattori who designed this study and who would perform the analysis.

### 2.2. MTB Diagnosis, Drug Susceptibility, Laboratory Test, and Chest Radiography

Acid-fast bacilli (AFB) smear staining and culture tests were performed to confirm MTB infection. Drug susceptibility test (DST) for isoniazid (INH), streptomycin (SM), rifampicin (RMP), and ethambutol (EMB) was conducted based on WHO-approved methods [28]. Laboratory data, including CRP, blood IgG, and IgA, were measured at Fukujuji Hospital. Chest radiographic data were read and summarized by three clinicians who had no access to other test results (Table 1).

### 2.3. Spoligotyping

The genotype of MTB clinical isolates was determined as described previously [29]. Briefly, the direct repeat (DR) region was amplified with a primer pair, and the PCR products were hybridized to a set of 43 spacer-specific oligonucleotide probes, which were covalently bound to the membrane. The spoligointernational type (SIT) was determined by comparing the spoligotypes against the international spoligotyping database (SpolDB4) [30].

### 2.4. Antigens

Gene cloning, expression, and purification method for Rv0679c was described previously [11]. Rv0679c142Asn and Rv0679c142Lys genes were cloned with the genomic DNA from non-Beijing and Beijing genotype MTB, respectively. The genes encoding Ag85A, Ag85B, ACR, HBHA, and MDP-1 were amplified by PCR from the genomic DNA of MTB H37Rv, and the primers were designed to incorporate restriction sites for cloning. The amplified DNA sequences were cloned into the pET-42 (+) vector (Novagen, South Africa), and the various vectors were expressed using* Escherichia coli* BL21 (DE3) (Novagen, South Africa). The recombinant proteins were purified via immobilized metal ion affinity chromatography using a nickel-sepharose high-performance column, according to standard protocols. The purity of the protein was analyzed by SDS-PAGE. The purified Lipoarabinomannan (LAM) (Nacalai Tesque, Kyoto, Japan) was isolated from MTB AoyamaB [31]. The purified trehalose methoxy-dimycolate (TDM-M) and trehalose keto-dimycolate (TDM-K) were semisynthesized by chemically introducing methoxy-mycolic acid and keto-mycolic acid, respectively, to the 6,6′ position on trehalose.

### 2.5. ELISA

To detect the antibodies against dimorphic surface protein Rv0679c in MTB, Nunc MaxiSorp plates (Thermo Fisher Scientific, Inc., Waltham, MA) were coated with 100 *μ*L of 0.2 *μ*g/mL purified recombinant Rv0679c142Asn or Rv0670c142Lys. Coated microplates were then allowed to stand overnight at 4°C, which were then blocked with 3% (w/v) bovine serum albumin (BSA) in phosphate-buffered saline (PBS; pH 7.4) for 4 h. Plasma samples were diluted by 100 times in 1% (w/v) BSA/PBS and then were added along with positive controls to set of triplicate wells. The positive controls were made of supernatant of mouse hybridoma cell culture that contained monoclonal antibody (mAb) 5D4-C2, which was diluted in 1/50-, 1/200-, and 1/800-fold dilution in 1% BSA/PBS [11]. The plates were sealed and incubated for 2 h at room temperature. After being washed 4 times with PBS-0.05% Tween 20 (PBST), 100 *μ*L of 1 : 10,000 times diluted HRP-conjugated goat anti-human IgG heavy chain polyclonal antibody (LifeSpan BioSciences, Inc., Seattle, WA) and 100 *μ*L of 1 : 10,000 times diluted HRP-conjugated goat anti-mouse IgG antibody (Santa Cruz Biotechnology, Inc., Dallas, TX) in 1% BSA/PBS were used as the second antibody for plasma samples and the mAb positive control, respectively. The sealed plates were then incubated for 2 h at room temperature. Then, the plates were washed 4 times and developed readable signal by 100 *μ*L of tetramethylbenzidine substrate (Dojindo, Kumamoto, Japan). To stop the reaction, 100 *μ*L of 0.5 M sulfuric acid was added to each well, and the absorbance was read at 450 nm. For other antigens, after 100 *μ*L of each of 4 *μ*g/mL HBHA, 14 *μ*g/mL Ag85A, 6 *μ*g/mL Ag85B, 1 *μ*g/mL ACR, 8 *μ*g/mL MDP-1, 3 *μ*g/mL TDM-M, 3 *μ*g/mL TDM-K, and 0.5 *μ*g/mL LAM were coated, similar methods were conducted to detect their specific corresponding IgG [25, 26]. Plasma TBGL IgG and TBGL IgA levels were measured using the* Determiner TBGL Antibody ELISA Kit* (Kyowa Medex Co., Ltd., Tokyo, Japan) [32].

### 2.6. Statistical Analysis

TB patients were grouped into Beijing and non-Beijing groups according to the results of spoligotyping. GraphPad Prism 6.0 (GraphPad Software, San Diego, CA) was used to analyze data and generate graphs. Comparison of clinical findings was achieved by nonparametric *t*-test and Chi-square test. Kruskal–Wallis test was used to evaluate differences in cases involving Beijing, non-Beijing, and HC groups. Dunn's post hoc tests were used to evaluate the differences between each pair following the Kruskal–Wallis test. Correlation analysis was accomplished by using Spearman correlation. The results were considered significant at *p* < 0.05. The cutoff for anti-TBGL antibodies was set to 2 on the basis of previous studies [32]. The cutoffs for other antibodies were achieved by Youden's index by MedCalc (MedCalc Software bvba, Belgium).

## 3. Results

### 3.1. Genotypes of MTB as Identified by Spoligotyping

Spoligotyping data elucidated the phenotype of 36 clinical MTB strains, among which the Beijing genotype MTB accounted for 20 cases (55.6%), while the non-Beijing genotype MTB accounted for 16 cases (44.4%) (Table 1). Multiple drug resistance (MDR) MTB shows resistance to RMP and INH in T1 strain from non-Beijing MTB group. None of strains from Beijing/Non-Beijing group were resistant to SM and EMB. Although the non-Beijing genotype group consisted of 6 MTB subtypes, there was no significant difference in the response to each antigen in terms of subtypes (Table 2).

### 3.2. Rv0679c ELISA

**Figure 1:**
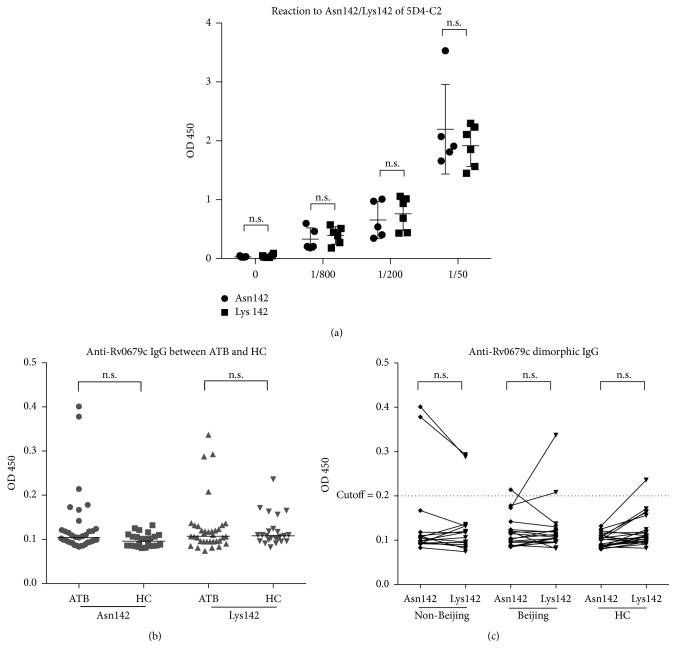
Antibody responses to Rv0679c142Asn and Rv0679c142Lys. (a) Anti-Rv0679c response observed at different dilutions of monoantibody 5D4C2 (0-, 1/50-, 1/200-, and 1/800-fold). (b) Anti-Asn142/Lys 142 IgG between the ATB and HC. Medians are indicated as lines. (c) Plasma antibody reaction to Rv0679c; 2 dots connected by a single line indicate a pair of reactions to Rv0679cAsn142 or Lys142 in one sample. The cutoff was set as twice the median of HC. n.s., not significant.

The ELISA test was optimized with regard to antigen concentration by using the monoclonal antibody 5D4-C2, which binds to both Asn142 and Lys142 without significant difference (Figure 1(a)). The ELISA assay for anti-Rv0679c142Asn IgG and anti-Rv0679c142Lys IgG showed that the majority in both non-Beijing and Beijing groups failed to react to each type of Rv0679c (Figure 1(b)), except for 2 samples in the non-Beijing group that showed higher reactivity to Rv0679c142Asn than Rv0679c142Lys and the 2 samples in the Beijing group that showed higher reactivity to Rv0679c142Lys than Rv0679c142Asn (Figure 1(c)).

### 3.3. IgG Antibodies against Ag85B, Ag85A, ACR, HBHA, TDM-M, TDM-K, MDP-1, and LAM

**Figure 2:**
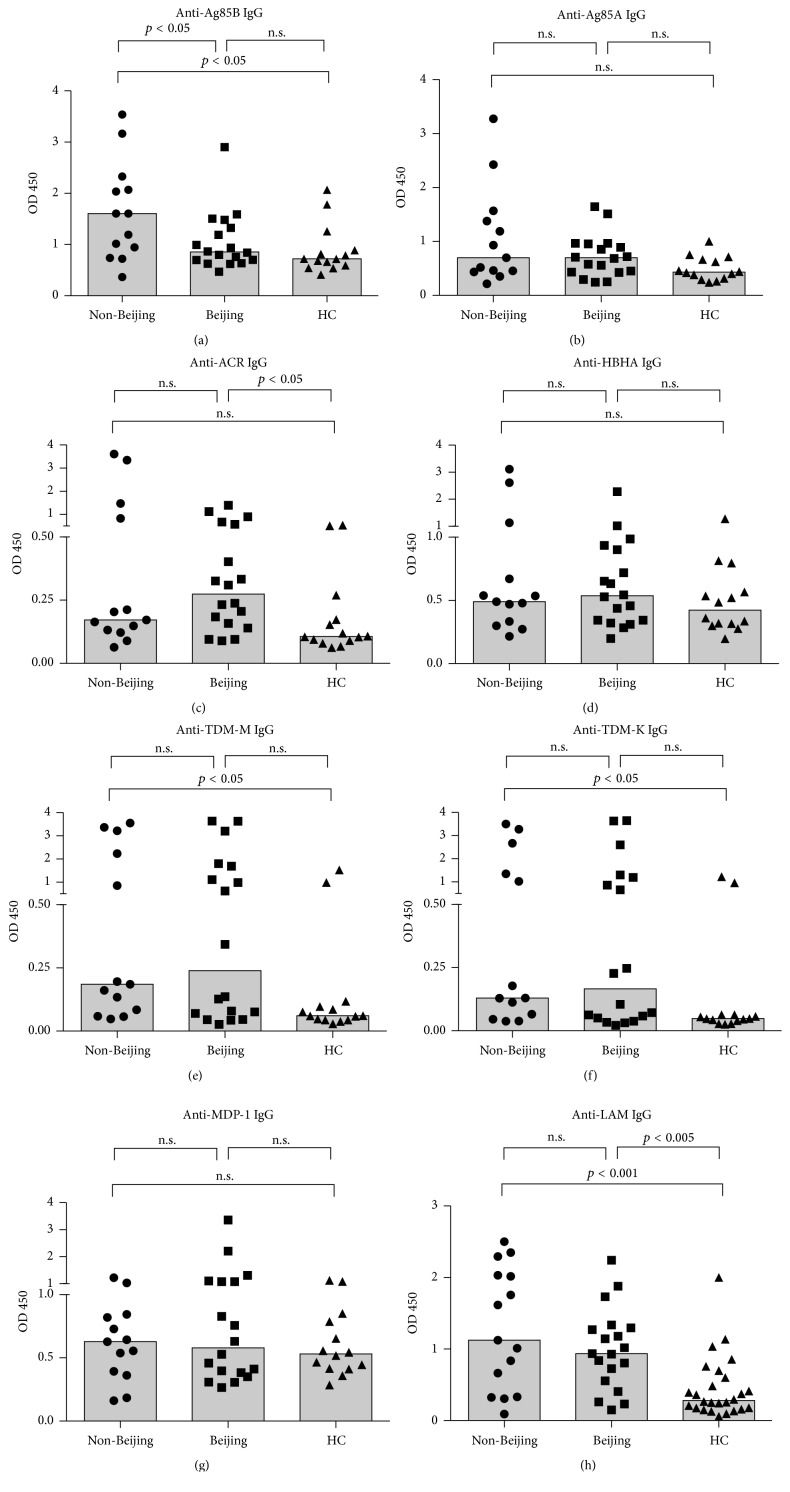
IgG antibody responses to Ag85B, Ag85A, ACR, HBHA, TDM-M, TDM-K, MDP-1, and LAM. (a) Anti-Ag85B IgG; (b) anti-Ag85A IgG; (c) anti-ACR IgG; (d) anti-HBHA IgG; (e) anti-TDM-M IgG; (f) anti-TDM-K IgG; (g) anti-MDP; (h) anti-LAM IgG responses. *p* value < 0.05 (Kruskal–Wallis test) indicated significant difference in the antibody response of the indicated groups. n.s., no significant. Medians are indicated as bars.

Of these 8 IgG antibodies, high titers of anti-Ag85 IgG were observed in the non-Beijing group compared to the Beijing group (Figure 2(a)). The comparison of IgG responses among non-Beijing, Beijing, and HC groups showed that the higher levels of anti-Ag85B IgG, anti-TDM-M IgG, and anti-TDM-K IgG were found in the non-Beijing group compared to the HC group (Figures 2(a), 2(e), and 2(f)). The anti-ACR IgG was higher in the Beijing group compared to the HC group (Figure 2(c)), while higher titers of anti-LAM IgG were found in both non-Beijing and Beijing groups compared to the HC group (Figure 2(h)). However, no significant difference was observed in the anti-Ag85A (Figure 2(b)), anti-HBHA (Figure 2(d)), and anti-MDP-1 (Figure 2(g)) IgG responses among the 3 groups.

### 3.4. Anti-TBGL Antibodies

**Figure 3:**
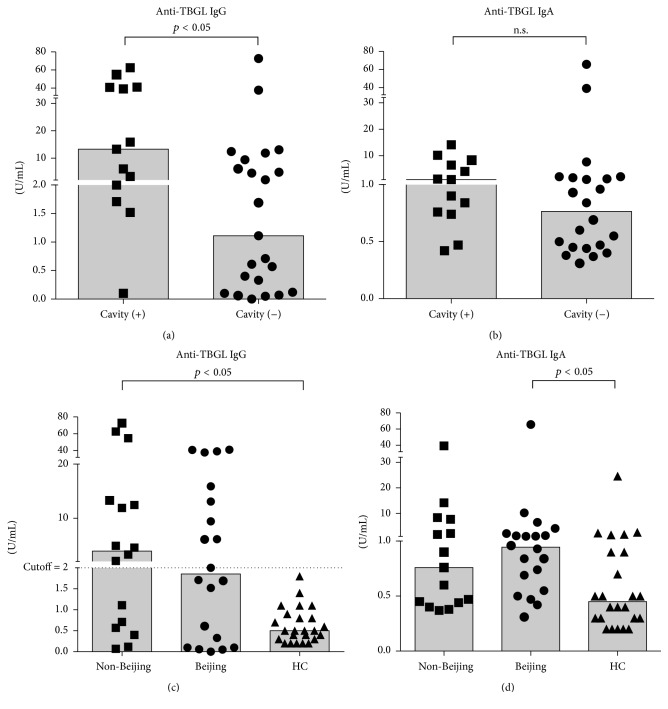
Association of anti-TBGL IgA and IgG responses and the presence of cavities and MTB genotype. *p* value < 0.05 indicated statistically significant difference. The cutoff of anti-TBGL IgG response is indicated in the instruction manual provided with the anti-TBGL IgG kit; (a), (b) cavities observed on chest radiographs; (c), (d) responses in non-Beijing and Beijing strain-infected individuals and HCs. Medians are indicated as bars.

The level of anti-TBGL IgG, rather than anti-TBGL IgA, was found to be higher in TB patients presenting with cavities (Figures 3(a) and 3(b)) as described elsewhere [32]. Despite the larger number of cavity-positive subjects in the Beijing group compared to the non-Beijing group (45.0% versus 26.7%, nonsignificant, Table 1), no difference was noted in the anti-TBGL IgG response between the Beijing and non-Beijing groups. Notably, anti-TBGL IgG showed higher titers in the non-Beijing and HC groups, but it did not differ in the Beijing group compared to the HC group (Figure 3(c)). On the other hand, anti-TBGL IgA was significantly higher in the Beijing group than in the HC group (Figure 3(d)). It is also noteworthy that all HCs were negative for anti-TBGL IgG.

### 3.5. Correlation Analysis

Significant correlations were observed between antibodies against lipid antigens and IgG against lipid-biosynthesis-related protein (Table 3). In detail, anti-Ag85B IgG titers correlated with antibodies against lipid TB antigens, including TDM-M/K, TBGL, and LAM. In contrast, anti-Ag85A IgG titers correlated with only anti-TBGL IgA and with anti-TDM-M. Anti-LAM IgG were found to be correlating with anti-TDM-M/K IgG and anti-TBGL IgA/IgG.

### 3.6. Sensitivity and Specificity

Receiver operating characteristic (ROC) curve analysis was used to compare the sensitivity and specificity of each TB antibody test. The cutoff was determined based on all TB subjects and HCs. An antibody test that met the following conditions, specificity > 60% and sensitivity > 50% either in the non-Beijing or in the Beijing group, at *p* < 0.05, was tentatively categorized as a prospective marker possessing discriminatory power, which includes anti-Ag85B IgG, anti-ACR IgG, anti-LAM IgG, and anti-TBGL IgG (Table 4). Of note, anti-Ag85B IgG showed higher response in the non-Beijing than in the Beijing group (76.9% versus 44.4%, Table 4), as opposed to anti-ACR IgG that showed higher response in the Beijing than in the non-Beijing group (61.1% versus 30.8%); both tests showed equal specificity up to 78.6%. High specificity was observed for anti-TBGL IgG and anti-LAM IgG, up to 100% and 83.3%, respectively. Combining anti-TBGL IgG and anti-LAM IgG increased the sensitivity up to 80% for the non-Beijing and 73.7% for the Beijing groups. Because anti-TBGL IgG were significantly higher in non-Beijing than in HC group, as well as higher anti-TBGL IgA in Beijing than in HC group, a test using the intersection of anti-TBGL IgG and anti-Ag85B IgG can yield higher accuracy to exclude the Beijing group within ATB (specificity, 77.8%), while the union of anti-TBGL IgA and anti-ACR IgG can yield higher accuracy to detect the Beijing group within ATB (sensitivity, 75.0%).

## 4. Discussion

In this study, high anti-ARC IgG and anti-TBGL IgA in Beijing TB infected patients, as well as high anti-Ag85B IgG and anti-TBGL IgG in Non-Beijing patients, could offer a sensitivity of 75.0% and a specificity of 77.8% for detecting Beijing MTB infection in diagnosed ATB. This can compensate the shortcoming of molecular diagnosis which needs bacteria derived DNA.

A mutated gene encoding the Lys142 variant of the Rv0679c protein has been found in the clinical isolates of the Beijing genotype family members [12]. The change caused by the Asn142Lys substitution may result in a reduced immune binding in response to a change of pathogenicity [33]. In this study, we observed a few specific recognitions of Rv0679c142Asn and Rv06790cLys142 antigens in the non-Beijing and Beijing groups, respectively, indicating discriminatory power upon IgG reactions against the distinct protein dimorphic between clades (Figure 1(c)). However, the reactions to MTB surface protein Rv0679c were not observed in the majority of samples (Figure 1(b)), agreeing with previous studies that found serological responses to MTB surface protein are relatively lower compared to those to cell wall component (LAM, e.g.,) or secreted proteins [34]. Active TB infected (ATB) patients may lack high titer-high avidity protective antibodies against the surface of MTB [10, 34].

Previous proteomics studies found that the expression of Ag85B appeared lower in Beijing genotype MTB in vitro [16, 17]; therefore, lower titers of anti-Ag85B IgG in the Beijing group than in the non-Beijing group (Figure 2(a)) may indicate lower burden of Ag85B during Beijing MTB infection [35]. Out of expectation, as Ag85B leads to the synthesis of TDM, we observed higher IgG against TDM-M, TDM-K, and TBGL in the non-Beijing group compared to the HC group (Figures 2 and 3), in addition to the significant correlation between anti-Ag85B IgG and anti-TDMs IgG (anti-TDM-M IgG, *p* < 0.05; anti-TDM-K and anti-TBGL IgG, *p* < 0.005; Spearman correlation). For the first time, we reported higher tier of anti-Ag85B IgG in the non-Beijing group (OD median 1.605; positivity 76.9%) compared those in the Beijing group (OD median 0.854; positivity 44.4%). Since the stronger induction of anti-Ag85B IgG resulted in better protective effects in ATB [36], higher burden of Ag85B and the corresponding higher levels of anti-Ag85B IgG may confer to more protective immune responses during non-Beijing MTB infection rather than did Beijing MTB. Unlike Ag85B, Ag85A plays a role in support of the growth of MTB under nutritional stress, which is crucial for the persistence of MTB in macrophage cells. Lacking the antibody responses against Ag85A during active TB infection may be in association with the immune escape due to the fact of MTB intracellular infection [13], in contrast to elevated anti-Ag85A IgG that were found in individuals recovered from TB [37].

Higher IgG response against ACR was observed in the Beijing group compared to the HC group (Figure 2(c)), which is consistent with the finding of higher expression of ACR protein in the Beijing strains in vitro [16]. However, no significant difference was noted in the anti-ACR IgG titer between the Beijing and non-Beijing groups. Meanwhile, as the marker of extrapulmonary TB [38], anti-HBHA IgG were not found to be different among the three groups (Figure 2(d)), which can be explained by the risk factors for extrapulmonary TB infection that are related more to host than to MTB strain lineage characteristics [39].

Anti-LAM IgG responses were observed in patients with smear-positive, culture-positive sputum [40, 41]. In spite of a significant correlation between anti-TBGL IgG and anti-LAM IgG, higher anti-TBGL IgG titers were observed in the non-Beijing group than in the HC group (*p* < 0.001). Meanwhile, significantly elevated anti-LAM IgG titers were observed in both the non-Beijing and Beijing group compared to the HC group, which was also reported in another study [35]. On the other hand, the anti-TBGL IgA titer in the Beijing group, instead of the non-Beijing group, appeared higher than those in the HC group. Of note, detecting anti-TBGL IgG is still practiced in TB diagnosis. TBGL mainly consists of TDM; therefore, the low anti-TBGL IgG levels in the Beijing genotype MTB group may be attributed to the small quantity of TDM that is associated with the low Ag85B expression in the Beijing group (Table 3). In our previous study in Indonesia where non-Beijing type MTB is prevalent up to 66.6% [42], 66.8% sensitivity of anti-TBGL IgG in detection of ATB was observed [26], which is higher than 55.6% sensitivity of that in this study where Beijing type TB infection made up a large proportion, suggesting anti-TBGL IgG titer be impacted by the infecting genotype of MTB. Moreover, in this study we found that all T-SPOT negative HC to be anti-TBGL IgG negative, while high anti-TBGL IgG were found in health care workers at the high risk of LTBI, indicating an association between anti-TBGL IgG and LTBI status [43]. In spite of the fact that limited samples are involved in this study, since anti-TBGL IgG is being used clinically irrespective of the MTB genotype the awareness is in need.

In a previous study, it has been observed that all the 7 individuals from the* Haarlem* MTB lineage-infected group showed positive reactions against TB Rv0407 antigen while only 2 out of 9 individuals from* Latin-American-Mediterranean* MTB lineage-infected group showed such positive reaction [44]. Meanwhile, in another study, anti-Esat6 IgG, anti-CFP10 IgG, and anti-LAM IgG responses were reported to be similar between Beijing and non-Beijing strain infection [35]. In conjunction with our result that anti-Ag85B IgG titers are higher in non-Beijing infection, these results suggest that the cornerstone for the genotype diagnosis method based on serologic responses should be built upon sophisticated screening for genotype-specific antigens. Therefore, a large number of TB antigens together with more plasma samples should be involved in future study.

## 5. Conclusions

In this study, we focused on serological antibody reactions against 11 antigens in terms of Beijing or non-Beijing genotype MTB infections by using a double-blind measure. IgG antibodies against Ag85B and TDM derivatives, including TDM-M, TDM-K, and TBGL, were found to be higher in non-Beijing genotype MTB infections compared to HC group, while anti-ACR IgG and anti-TBGL IgA were found to be higher in the Beijing compared to HC group. Despite the fact that out of 11 antigen-antibody tests only anti-Ag85B IgG showed higher response in non-Beijing TB patients than in Beijing TB patients (*p* < 0.05), the combination of anti-Ag85B IgG, anti-ACR IgG, and anti-TBGL antibodies tests could yield 75.0% sensitivity and 78.8% specificity for detecting Beijing MTB infection in ATB individual. Therefore, discrepancy in the antibody responses in the Beijing and non-Beijing groups could aid the identification of Beijing genotype MTB-specific infections.

## Figures and Tables

**Table 1:** Clinical characteristics.

	Non-Beijing	Beijing	*p* value
	*n* = 16	*n* = 20	
Demographics			
Males, *n* (%)	14 (87.5%)	14 (70.0%)	n.s.
Age [y, mean (%)]	50.7	60.2	n.s.
Mycobacterial identification			
AFB-positive strains, *n* (%)	13 (81.2%)	17 (85.0%)	n.s.
Time to growth in MGIT (weeks)	7.43	8.20	n.s.
Drug resistance profile			
Isoniazid-resistant strain	0	3 (15%)	n.s.
MDR strain	2 (12.5%)	0	n.s.
Laboratory findings			
CRP (mg/mL)	6.55 ± 4.38	5.84 ± 4.38	n.s.
Blood IgG (mg/mL)	17.67	16.12	n.s.
Blood IgA (mg/mL)	3.83	3.99	n.s.
Chest radiograph findings			
Cavity, positive (%)	4 (26.7%)	9 (45.0%)	n.s.
Pleural effusion, positive (%)	3 (20.0%)	5 (25.0%)	n.s.

STD, standard deviation; MDR, multidrug resistance, as resistant to rifampin and isoniazid in this study; CRP, C-reactive protein.

**Table 2:** Non-Beijing genotype subgroups.

	Subtypes of non-Beijing MTB					
Genotype	EAI2 Manila	LAM9	New type	T1	T2	T3-OSA
	*n* = 2	*n* = 1	*n* = 5	*n* = 5	*n* = 1	*n* = 2
Number of MDR strains (%)	0	0	0	2 (40%)	0	0
Anti-LAM IgG^a^	1.34 (0.33–2.35)	1.01	0.84 (0.09–2.5)	1.37 (0.33–2.29)	0.310	1.89 (1.76–2.03)
Anti-TBGL IgG^a^	2.71 (2.11–3.30)	12.44	0.57 (0.07–11.9)	34.1 (0.12–62.6)	1.11	38.8 (4.9–72.6)
Anti-TBGL IgA^a^	7.28 (0.37–14.2)	0.45	0.54 (0.44–39.2)	0.83 (0.38–8.38)	2.13	4.7 (1.72–77.0)
Anti-Ag85B^a^	1.31 (1.01–1.61)	0.37	1.17 (0.72–3.16)	1.61 (0.94–2.32)	3.54	2.07
Anti-ACR^a^	0.15 (0.12–0.17)	0.15	0.15 (0.09–0.16)	0.21 (0.06–1.47)	3.35	3.61

^a^Median (range), EAI 2 Manila Clade EAI2 from the Manila family strain; LAM9, *Latin *American-Mediterranean; T1, specific T1 genotype clone (SIT number 266); T2, T2 *Mycobacterium tuberculosis* genotype.

**Table 3:** Correlation analysis.

	Anti-Ag85B IgG		Anti-TDM-M IgG		Anti-TDM-K IgG		Anti-Ag85A IgG		Anti-TBGL IgG IgG		Anti-TBGL IgA IgG		Anti-LAM IgG	
	*r*	*p*	*r*	*p*	*r*	*p*	*r*	*p*	*r*	*p*	*r*	*p*	*r*	*p*
Anti-Ag85B IgG			0.42	*∗*	0.44	*∗*	0.59	*∗∗∗*	0.52	*∗∗*	0.45	*∗*	0.58	*∗∗∗*
Anti-TDM-M IgG	0.42	*∗*			0.99	*∗∗∗*	0.36	*∗*	0.80	*∗∗∗*			0.77	*∗∗∗*
Anti-TDM-K IgG	0.44	*∗*	0.99	*∗∗∗*					0.83	*∗∗∗*			0.79	*∗∗∗*
Anti-Ag85A IgG	0.59	*∗∗∗*	0.36	*∗*							0.37	*∗*		
Anti-TBGL IgG IgG	0.52	*∗∗*	0.80	*∗∗∗*	0.83	*∗∗∗*							0.94	*∗∗∗*
Anti-TBGL IgA IgG	0.45	*∗*					0.37	*∗*					0.60	*∗∗∗*
Anti-LAM IgG	0.58	*∗∗∗*	0.77	*∗∗∗*	0.79	*∗∗∗*			0.94	*∗∗∗*	0.60	*∗∗∗*		

*p* values of Spearman's rank correlation are expressed as follows: ^*∗*^*p* less than 0.05, ^*∗∗*^*p* less than 0.01, and ^*∗∗∗*^*p* less than 0.001.

**Table 4:** Discriminatory power analysis.

TB antibodies	Cutoff^a^	Sensitivity (%)			Specificity (%)	*p *value
		TB subjects	Non-Beijing	Beijing	HC	
Anti-Rv0679c142Asn IgG	0.09	83.1	93.3	75.0	45.8	**0.0301**
Anti-Rv0679c142Lys IgG	0.10	65.6	60.0	70.0	29.2	n.s.
Anti-Ag85B IgG	0.89	58.2	76.9	44.4	78.6	**0.035**
Anti-Ag85A IgG	0.76	42.0	46.2	38.9	92.9	**0.0151**
Anti-ACR IgG	0.21	48.2	30.8	61.1	78.6	**0.0024**
Anti-HBHA IgG	0.82	25.8	23.1	27.8	92.9	n.s.
Anti-TDM-M IgG	0.50	41.9	38.5	44.4	85.7	**0.0025**
Anti-TDM-K IgG	0.53	38.7	38.5	38.9	85.7	**0.0031**
Anti-MDPI IgG	0.40	67.8	69.2	66.7	14.3	n.s.
Anti-LAM IgG	0.76	67.7	66.7	68.4	83.3	**<0.0001**
Anti-TBGL IgG (U/mL)	2.00^b^	55.6	62.5	50.0	100.0	**0.0006**
Anti-TBGL IgA (U/mL)	1.00	42.9	40.0	45.0	80.1	**0.0008**
Anti-TBGL IgG (U/mL) or anti-LAM IgG	—	76.5	80.0	73.7	83.3	—
Anti-TBGL IgG (U/mL) and anti-Ag85B IgG	—	35.6	53.8	22.2	100.0	—
Anti-ACR IgG or anti-TBGL IgA (U/mL)	—	59.4	40.0	75.0	64.3	—

The sensitivity of the test to discriminate between non-Beijing and Beijing genotype TB was calculated in the context of the cutoff values obtained for all TB subjects. A *p* value less than 0.05 indicates significant discriminatory power.

^a^Optimal cutoff was determined based on the ROC curve obtained for TB patients and healthy controls according to Youden's index.

^b^Referring to the recommended cutoff, in accordance with the instructions of the anti-TBGL IgG kit.
